# The Belgian commitment to pharmaceutical quality: a model policy to improve quality assurance of medicines available through humanitarian and development programs

**DOI:** 10.1186/s40545-018-0136-z

**Published:** 2018-04-19

**Authors:** Raffaella Ravinetto, Tim Roosen, Catherine Dujardin

**Affiliations:** 1 Institute of Tropical Medicine Antwerp, Antwerp, Belgium; 2 Be-cause Health (Belgian platform for international health), Antwerp, Belgium; 3 Belgian Directorate-General for Development Cooperation and Humanitarian Aid, Brussels, Belgium

**Keywords:** Quality of medicines, Quality assurance, Belgium, Low-income countries, Capacity-building, Pharmaceutical policy, Supply

## Abstract

Today, a combination of globalization of pharmaceutical production, lack of regulatory harmonization, and weakness of Medicines Regulatory Authorities, creates the “perfect conditions” for poor-quality medicine to circulate in the global market and to penetrate the less-regulated countries. Medicines regulation is the responsibility of the national regulatory authorities in the recipient country, but in the poorer countries, in practice, the responsibility of supply of quality-assured medicines is often taken by Non-Governmental Organizations and other implementers. But with some notable exceptions, many donors lack a pharmaceutical procurement policy with adequate quality requirements; and many implementers lack the skills and expertise needed to orient themselves in the complex web of global pharmaceutical supply. Thus, patients served by humanitarian or development programs may remain exposed to the risk of poor-quality medicines.

When public money is used to purchase medicines for medical programs to be carried out overseas, adequate policies should be in place to assure that the same quality requirements are set that would be required for medicines marketed in the “donor” country.

We will describe here a policy recently adopted in Belgium, i.e. the “Commitment to Quality Assurance for Pharmaceutical Products”, signed in October 2017 by the Vice Prime Minister and Minister for Development Cooperation and 19 Belgian implementing agencies. By signing the new policy, the counterparts committed to ensure quality of medicines in the programs funded by Belgium’s Official Development Assistance, and to build quality-assurance capacity in the recipient countries. Implementers are requested to integrate in their financing applications a section for pharmaceutical quality assurance, with a justified budget. They are also invited to consider how costs could be rationalized and mutualized by aligning the strengths of the various implementers. This model policy has the potential to be considered for adoption by other donors, to help to reduce the current multiple standards in pharmaceutical quality, and to contribute to protect vulnerable communities from the plague of poor-quality medicines.

**Electronic additional files:**

The online version of this article (10.1186/s40545-018-0136-z) contains an additional file, which is available to authorized users.

## Quality challenges in low-income countries

Poor-quality medicines harm individuals, who either don’t get cured or become victims of potentially serious adverse events; they harm public health, by contributing to resistances to anti-infective medicines; and they harm health systems, by eroding public trust in medicine and causing waste of resources [1, 2]. The “perfect conditions” for poor-quality medicines to penetrate the poorer, less-regulated countries are created by an unfortunate combination of globalization of production/distribution, lack of global regulatory harmonization, erratic supply, weakness of National Medicines Regulatory Authorities (NMRAs) in many low-income countries (LICs), and complexity of the supply chains [3–6]. The important body of evidence that poor-quality medicines are massively present in LICs [7–9], may be just the top of the iceberg: according to the World Health Organization (WHO), the number of reports of substandard and falsified medical products heavily depend on who is looking out for them, and on whether they know how and where to report [10].

## Influence of donors and NGOs in low-income countries

Medicines regulation is the responsibility of the NMRAs in the recipient countries, and effective national regulatory systems are an essential component of health system strengthening [11]. The WHO supports NMRAs in different ways: the Expert Committee on Specifications for Pharmaceutical Preparation advises Member States on medicines quality assurance (QA) [12]; a Good Regulatory Practices guideline is under preparation [13]; the Prequalification (PQ) Programme orients purchasers toward quality-assured products for some selected conditions, and enables NMRAs to strengthen their oversight processes, through a collaborative procedure [14].

Nonetheless, in most LICs the NMRAs are still under-resourced, i.e. they lack adequate financial and human resources, as well as adequate infrastructure such as functional national Quality Control laboratory, to carry out their task [5]. These countries also largely depend on external funds for a great part of their pharmaceutical supply, so that the supply of quality-assured medicines is often in practice the responsibility of donors, Non-Governmental Organizations (NGOs) and other implementers. Through their procurement choices, they de facto determine the quality of medicines distributed through public and humanitarian sectors. Social enterprise medicines traders (e.g. charities wholesaling medicines, international NGOs distributing medicines, etc.) may exercise an even broader informal regulatory influence [15]. But are adequate QA requirements always explicitly spelled out by humanitarian and development agencies, and by donors?

The procurement policies of some major NGOs and donors take in due account the risks inherent to international market, and require compliance with adequate specifications. This is for instance the case of Médecins Sans Frontières [16, 17] and the Global Fund [18], while the European Civil Protection and Humanitarian Aid Operations (ECHO) Directorate-General maintains a register of approved procurement centers [19]. But to the best of our knowledge, many donors lack a procurement policy with adequate quality requirements [20], and many implementers lack the skills and expertise to orient themselves in the global pharmaceutical supply. Donors and implementers often dedicate insufficient financial and human resources to QA, due to lack of awareness or of political will. Thus, patients served by humanitarian or development programs may remain exposed to the risk of poor-quality medicines.

Universal access to quality-assured essential medicines is a determinant of the fulfilment of the right to health [21], and humanitarian or development agencies should play an active role to achieve it. In particular, when public money is used to purchase medicines for medical programs overseas, all possible efforts should be done to avoid differences in the level of QA (and thus, of protection) for patients in the “donor” and in the “beneficiary” country. In other words, as stated in the WHO Guidelines for Medicines Donations, “there should be no double standard in quality. If the quality of an item is unacceptable in the donor country, it is also unacceptable as a donation” [22]. But translating this principle into practices is not obvious, since quality risks are usually much higher in low-income, “beneficiary” countries, compared to the high-income “donor” countries [3, 4, 10], and since there is still insufficient awareness of the importance to avoid double standards (for instance, there is broad evidence of well-intended medicine donations that have caused problems instead of bringing relief [22]).

## A Belgian commitment to quality-assured medicines for all

The Belgian Directorate-General for Development Cooperation & Humanitarian Aid (DGD) is the institutional donor and main contributor to Belgium’s Official Development Assistance (ODA). Belgium’s ODA is predominantly active in 14 partner countries, mainly in Africa. About 10% of its programs are in the health sector, with a yearly budget of 115,6 million euro (in 2016). Overall, about 22% of financial resources in the health sector are allocated to the purchase of medicines [23], via 19 implementers/purchasers. The DGD has been confronted to quality-related challenges through the experience of its implementers, and it has been looking for an adequate response by engaging in dialogue with them. This bottom-up process involved relevant Belgian implementers and Belgian federal public services (such as Finance, Public Health and Foreign Affairs), within the framework of policy coherence for sustainable development. Inputs were sought from the WHO and the European Commission. The dialogue was facilitated by the fact that most implementers are active in Be-cause Health, an informal and pluralistic Belgian platform that provides a place for exchange and capitalisation of technical knowledge and scientific evidence on international health and development cooperation [24]. Be-cause Health also provides a specific space (“working group”) that brings together individuals and organizations interested/involved in the management of medicines in the context of international health and development cooperation [25].

The process resulted in a “Commitment to Quality Assurance for Pharmaceutical Products”, signed on 25th October 2017 in Brussels by the Belgian Deputy Prime Minister and Minister for Development Cooperation Alexander De Croo and by 19 Belgian implementers, i.e. NGOs, Belgian development agency, academia, and the Belgian investment company for developing countries [26, 27]. With this Commitment (English version available as Additional file 1), the Belgian State, as an institutional donor country, and as part of its contribution to the implementation of the Sustainable Development Goals, commits to manage the quality risks of medicines in programs funded by Belgian ODA; and the Belgian implementers commit to strive to supply quality-assured medicines to the recipients of ODA-funded programs.

In line with the Minister’s general policy, the implementers should be results-oriented, and they bear the final responsibility to adequately implement the **C**ommitment. In particular, they should set up a QA system, that is, a process of pre-qualification of suppliers, purchasing, storage and distribution practices, and monitoring-evaluation, accompanied by risk analysis and management, and with full product traceability from the actual manufacturer to end users. The quality of procured medicines must be “acceptable”, i.e. in compliance with the standards of the WHO and/or the International Conference of Harmonization, and “measurable”, i.e. a “proof of quality” should be available, such as WHO pre-qualification, registration by a stringent regulatory authority [28], or (in their absence) evidence from a recent expert audit carried out by a qualified expert. These requirements may be read as an “extension” to *development* programs of the WHO principles for medicines’ donations (which mainly refer to *emergency* situations): “all donated medicines should be obtained from a quality-ensured source and should comply with quality standards in both donor and recipient countries” [22].

## Challenges: Costs, capacity building, poor understanding of gains

The initial stumbling block of the process was fear that the workload and the implementation costs would be unbearable or not manageable. Therefore, the Commitment integrates pragmatic administrative and financial rules. Implementers are requested to integrate in their financing applications a specific section for pharmaceutical QA, with a justified corresponding budget. They are also invited to consider how costs “could be rationalized and mutualized by aligning the strengths of the various implementers”. Concerns might remain about the costs of building a QA system, especially for those who must start from zero. However, an exclusive focus on the “direct costs” (such as hiring a QA pharmacist, developing a QA policy, sub-contracting pharmaceutical audits to qualified experts, upgrading storage facilities, etc.) would ignore the long-term gains of investing in QA, as well as the losses caused by not doing it: (undetected) poor-quality medicines would impose higher costs on implementers’ programs and local health systems, cause avoidable morbidity and mortality, and erode trust in medical services.

A second stumbling block was the fear that the process would result in the (further) weakening of the local pharmaceutical supply systems. To mitigate it, the “Commitment” recommends considering local purchases (provided that quality risks are assessed and mitigated), and prioritizing existing local structures for storage and distribution. Such choices should be based on risk analysis and management, and accompanied by plans to strengthen the local capacities, i.e. through capacity building for pre-qualification, purchases, storage and distribution, depending on needs (e.g. of local procurement agencies). The implementers should integrate in their financing applications a specific section for such activities, and consider if/how costs could be rationalized and mutualized. Noteworthy, academic institutions may contribute to capacity-building, by means of education programs and collaborative operational research [29]. This approach addresses the fears that the “Commitment” would overrule the power of the NMRAs, so contradicting the principle of state sovereignty, or indulging in paternalism. The NMRAs should be a privileged partner of DGD implementers to discuss any relevant issues, included but not limited to audits planned in-country, audits findings, medicines importation (that can only take place with the NMRA approval) and possible research collaborations.

When it comes to possible adoption of this “model policy” by other donors or implementers, a third stumbling block is the possibility that the potential gains of the policy are misunderstood. In particular, donors, implementers and also policy-makers may still be unaware of the potential detrimental effects of non-quality-assured products, which often go undetected. For instance, therapeutic failure caused by an under-dosed medicine can be attributed to other reasons, such as late referral, and even cases of toxicity due to a contaminated medicine may go unnoticed unless cases are clustered, e.g. at hospital level. But “treating” patients, i.e. administering a medicine the quality of which has not been adequately verified, is different from “curing” patients, i.e. administering a quality-assured medicine which has been proven to be effective and safe. We hope that more and more policy-makers, implementers and donors become aware that investing in quality assurance results in gains for *health* (improved quality of care), *ethical behavior* (no double standards between patients in affluent and poor countries) and even *cost-effectiveness* (better quality of care means less therapeutic failures, and decreased long-term health costs). In addition, if more donors and implementers joined forces in requiring quality-assured products, they would create a “market incentive” to quality that could in the long-term lead to broader availability and lower prices of such products (as shown by the experience of WHO pre-qualified antiretroviral and antimalarial). A risk of blockage would remain, in case local suppliers were unable to supply quality-assured products and it was impossible to import. To mitigate this risk, it is essential to involve the key-local counterparts and especially the NMRAs in the development of the strategic approach, with the ultimate aim of empowering local partners and maximizing patients’ protection.

Finally, acknowledging the complexity of this undertaking, the new policy will be implemented in a stepwise approach, by means of a flexible and constructive monitoring and evaluation, with ongoing “peer-reviews” (via Be-cause Health) and concerted corrections between implementers and the DGD. In the long term, the step-wise approach could lead to defining criteria for accreditation of implementers.

## Conclusion

By signing the “Commitment to QA for Pharmaceutical Products”, Belgium commits itself as donor country to the quality of medicines provided in development and humanitarian programs, and it encourages national implementers to move beyond the immediate hurdles, for embracing the long-term human gains of investing in QA. This initiative should be evaluated in the middle- and long-term in terms of feasibility, cost-effectiveness, costs rationalization, and capacity building. Concomitantly, this model policy could be considered, with any relevant adaptations, by other public or private donors, for ensuring the quality of medicines supplied under their financing, and for contributing to protect vulnerable communities from poor-quality medicines.

## Additional file

Additional file 1: Supplementary data. (PDF 137 kb)

## Abbreviations

DGD: Belgian Directorate-General for Development Cooperation & Humanitarian Aid
LICs: Low Income Countries
NGOs: Non-governmental Organizations
NMRA: National Medicines Regulatory Authority
ODA: Belgium’s Official Development Assistance
PQ: Pre-qualification Programme
SSFFC: Substandard, spurious, falsely labelled, falsified and counterfeit
WHA: World Health Assembly
WHO: World Health Organization

## References

[1] World Health Organisation. Substandard and falsified medical products. 70th World Health Assembly; 2017 22-31 May; Geneva, Switzerland [cited 2017 Nov 5]. Available from: http://www.who.int/mediacentre/news/releases/2017/dementia-immunization-refuguees/en/

[2] Ravinetto R, Vandenbergh D, Macé C, Pouget C, Renchon B, Rigal J, Schiavetti B, Caudron JM (2016). Fighting poor-quality medicines in low- and middle-income countries: the importance of advocacy and pedagogy. J Pharm Policy Pract.

[3] Caudron JM, Ford N, Henkens M (2008). Substandard medicines in resource-poor settings: a problem that can no longer be ignored. Trop Med Int Health.

[4] Newton PN, Amin AA, Bird C, Passmore P, Dukes G, Tomson G (2011). The primacy of public health considerations in defining poor quality medicines. PLoS Med.

[5] World Health Organisation. Assessment of medicines regulatory systems in sub-Saharan African countries. World Health Organisation, 2010. Last accessed on 11/11/2017 at http://apps.who.int/medicinedocs/en/d/Js17577en/.

[6] Nebot Giralt A, Schiavetti B, Meessen B (2017). Quality assurance of medicines supplied to low-income and middle-income countries: poor products in shiny boxes?. BMJ Global Health.

[7] Nayyar G, Breman J, Newton P, Herrington J (2012). Poor-quality antimalarial drugs in southeast Asia and sub-Saharan Africa. Lancet Infect Dis.

[8] Almuzaini T, Choonara I, Sammons H (2013). *Substandard a*nd counterfeit medicines: a systematic review of the literature. BMJ Open.

[9] Johnston A, Holt DW (2014). Substandard drugs: a potential crisis for public health. Br J Clin Pharm.

[10] World Health Organization (WHO). WHO Global Surveillance and Monitoring System for substandard and falsified medical products. WHO/EMP/RHT/2017.01. WHO 2017. Geneva, Switzerland. ISBN: 978-92-4-151342-5

[11] World Health Assembly (WHA). Resolution WHA67.20. Regulatory system strengthening for medical products. In: Sixty-Seventh WHA, Geneva, 19-24 May 2014. Geneva: World Health Organization; 2014 (http://apps.who.int/gb/ebwha/pdf_files/WHA67/A67_R20-en.pdf, accessed 2 Feb 2016)

[12] Norms and standards (2017). 70 years of WHO standards on medicines quality. WHO Drug Information.

[13] World Health Organization (WHO) (2016). Good regulatory practices: guidelines for national regulatory authorities for medical products. Working document QAS/16.686. Draft for comment Prepared by EMP/RSS.

[14] Accelerated Registration of Prequalified Finished Pharmaceutical Products. Last accessed on 8/1/2018 at https://extranet.who.int/prequal/content/collaborative-procedure-accelerated-registration

[15] Mackintosh M, Chaudhuri S, Mujinja P (2011). Can NGOs regulate medicines markets? Social enterprise in wholesaling, and access to essential medicines. Global Health Biomed.

[16] MSF Medical Product Procurement. Last accessed on 8/1/18 at http://www.msf.org/en/article/msf-medical-product-procurement

[17] MSF Medical Product Qualification Scheme. Last accessed on 8/1/18 at http://www.msf.org/en/article/msf-medical-product-qualification-scheme

[18] Global Fund Policy. Available at https://www.theglobalfund.org/media/5894/psm_qapharm_policy_en.pdfhttps:/www.theglobalfund.org/media/5894/psm_qapharm_policy_en.pdf

[19] Register of Humanitarian Procurement Centres (HPCs) recognised by DG-ECHO. Last accessed on 8/1/2018 at http://ec.europa.eu/echo/files/partners/humanitarian_aid/HPC-register_en.pdf

[20] Perrin C. Quality assurance policy for the selection of medicines in place at three major international donors: The Global Fund, The World Bank & ECHO. Their impact on the quality of medicines procured with their funds in limited resource settings. Thesis submitted to obtain the degree of Master in Public Health-Disease Control Antwerp: Institute of Tropical Medicine, Antwerp, 2007. Available on request

[21] Hogerzeil HV (2006). Essential medicines and human rights: what can they learn from each other?. Bulletin of the World Health Organization.

[22] World Health Organization (WHO). Guidelines for Medicines Donation. WHO, Geneva. Third Edition 2011. ISBN 978 92 4 150198 9

[23] Belgian Directorate-General for Development Cooperation & Humanitarian Aid (DGD). Annual report 2016. Available at https://diplomatie.belgium.be/sites/default/files/fin_jv2016_en_web.pdf

[24] Be-cause Health. Belgian Platform for International Health, Access to Medicines Working Group. Last accessed on 8/1/2018 at https://www.be-causehealth.be/en/bchgroups/access-to-quality-medicines/.

[25] Be-cause Health. Belgian Platform for International Health, Access to Medicines Working Group. Recommendations on the quality, availability and affordability of reproductive health supplies. Last accessed on 8/1/2018 at https://www.be-causehealth.be/wp-content/uploads/2017/09/RHSupplies-seminar-08-11-Insights-Recommendations.pdf

[26] Minister De Croo and Belgian actors from the International Health Cooperation sign commitment on quality of medicines. Last accessed on 8/1/2018 at https://www.be-causehealth.be/en/bch-news/minister-de-croo-and-belgian-actors-from-the-international-health-cooperation-sign-commitment-on-quality-of-medicines/

[27] Commitment to quality assurance for pharmaceutical products, between the Belgian Development Cooperation and the actors involved in the implementation of programmes including the purchasing, storage, distribution and/or control of pharmaceutical products. Brussels, Belgium, 25 October 2017. Available at https://diplomatie.belgium.be/sites/default/files/downloads/commitment_to_quality_assurance_for_pharmaceutical_products.pdf.

[28] WHO Expert Committee on Specifications for Pharmaceutical Preparations. Technical Report Series (TRS) 1003 - Fifth first report. Who, Geneva, June 2017. ISBN: 978 92 4 121003 4

[29] Schiavetti B, Wynendaele E, De Spiegeleer B, Mbinze GJ, Kalenda N, Marini R, Melotte V, Hasker E, Meessen B, Ravinetto R, Van der Elst J, Ngeleka DM. The Quality of Medicines. Internal Democratic Republic of Congo: A Prospective Survey. Am J Trop Med Hyg 2018. Published online ahead of print. DOI: https://doi.org/10.4269/ajtmh.17-073227 10.4269/ajtmh.17-0732 PMC5930909 29313479

