# Supplementary material for: The Belgian commitment to pharmaceutical quality: a model policy to improve quality assurance of medicines available through humanitarian and development programs
Source: J Pharm Policy Pract. 2018 Apr 19;11:12. doi: 10.1186/s40545-018-0136-z (PMC5907286; doi:10.1186/s40545-018-0136-z)
Supplement: Supplementary file 1 — Supplementary data. (PDF 137 kb) [file 40545_2018_136_MOESM1_ESM.pdf]

Commitment to Quality Assurance for pharmaceutical products

BETWEEN

DE BELGIAN DEVELOPMENT COOPERATION

AND

THE ACTORS INVOLVED IN THE IMPLEMENTATION

OF PROGRAMMES INCLUDING THE PURCHASING, STORAGE,

DISTRIBUTION AND/OR CONTROL OF PHARMACEUTICAL PRODUCTS

BRUSSELS, 25 OCTOBER 2017

Preamble

The Belgian Development Cooperation, namely the **Directorate General for Development Cooperation and Humanitarian Aid** (**DGD**) of the FPS Foreign Affairs, Foreign Trade and Development Cooperation (hereinafter referred to as "the Administration"), represented by the Minister of Development Cooperation,

and

The **actors involved in the implementation of programmes financed by the Belgian Official Development Aid**, programmes including the purchasing, storage, distribution and/or control of pharmaceutical products[a] : the Belgian actors of Governmental cooperation, of Non-governmental cooperation (NGOs and Institutional actors) and Humanitarian aid, the Belgian Investment Company for Developing Countries (BIO) (hereinafter referred to as "the implementing actors")

Considering that Belgium is committed to the implementation of the Agenda 2030 for Sustainable Development, a target of which is devoted to quality essential medicines and vaccines (SDG3.8),

Considering that Belgium recognizes the right to health, in which access to quality essential pharmaceutical products should be guaranteed. Considering that Belgium accesses to the Constitution of the World Health Organization and to various resolutions of the World Health Assembly on pharmaceuticals. To this end, Belgium supports the strengthening of health systems, in which pharmaceutical products are intended to be available, with assured quality and affordable,

Considering national and international standards and regulations on the quality of pharmaceutical products issued by the World Health Organization and issued by the Federal Agency for Medicines and Health Products, the FPS Public Health and the European Directives - and the rules on public procurement. Considering that this Commitment has also received the visa of the Directorate General for Budget and Management Control of the FPS Foreign Affairs, Foreign Trade and Development Cooperation (B&B) and the Inspector of Finance,

Considering that many Belgian implementing actors have committed themselves to implementing the Be-cause Health's Charter for the quality of medical products, Charter which is considered a milestone at the Belgian level (2008).

Now, have agreed on the principles of political orientation to get the implementing actors to better respond to local needs. These principles take into account the following:

Article 1: Objectives

1. Belgium and the implementing actors **commit to ensure quality of pharmaceutical products** in the programs financed by Belgian Official Development Aid,
2. Belgium and the implementing actors **commit to strengthen local capacities** of the partner country's procurement system in order to guarantee quality of pharmaceutical products,
3. Belgium and the implementing actors **commit to supply pharmaceutical products of assured quality** to the recipients of programmes funded by Belgian Official Development Aid.

[a] Pharmaceutical products : all medications (including vaccines, biological products, therapeutic and diagnostic medical devices) and health products (preservatives, food supplements, impregnated mosquito nets, etc.).

Article 2: Responsibilities

The implementing actors are responsible for the quality of the pharmaceutical products they are purchasing, storing and distributing through their programmes/projects.

Article 3: Commitments of the implementing actors & monitoring-evaluation

1. **Integrate a specific section for** the quality assurance of pharmaceutical products with the **justified budget** that is to be allocated to it. Add a baseline to this section for a mid-term report and an end-of-programme/project report.

   Monitoring-evaluation: *Ex ante*, before accepting a financing application, the Administration verifies whether the financing application contains this section. In order to obtain the approval of the Administration, of the financial controllers of Administration and of the Inspector of Finance, the budget must be justified and acceptable.

2. **Develop and implement a quality assurance policy** (process of pre-qualification of suppliers, purchasing, storage and distribution, monitoring-evaluation) accompanied by a **risk analysis and management**. E stablish standard operational procedures, if necessary differentiated depending on the context (audits of the local markets and an adequate and up-to-date knowledge of the local legislation can help define it).

   Respecting this Commitment may require an initial investment for the implementing actor. Possible costs could be rationalized and mutualized by aligning the strengths of the various implementing actors.

   Monitoring-evaluation: During implementation and *ex-post* (e.g. during the concertation meetings with the implementation actors), the Administration carries out a monitoring and intermittent « *checks* » on samples, possibly with support from Be-cause Health (« *peer reviews* »). Concerted corrections are proposed.

3. **Follow the criteria listed below with regard to purchasing, storage, distribution and local capacity strengthening.**

   Possible costs involved in complying with this commitment must be justified and budgeted in advance in the financing request.

   Monitoring-evaluation: During implementation and *ex-post*, the financial controllers of the Administration check to insure that the dossiers contain the supporting documents that comply with the criteria. Reporting to the Administration.

4. Complying with these commitments requires resources and technical competences. The delegation of certain tasks (technical support for the audits, evaluations, tenders or assistance for the development and implementation of the quality assurance policy, etc.) is possible, knowing that the actor involved in the implementation retains the final responsibility.

Article 4: Criteria to be followed by the implementing actors

1. Criteria for an « acceptable and measurable quality » :

The quality of the pharmaceutical products must be acceptable and measurable.

a. Acceptable = **in compliance with the norms and standards of the World Health Organization** (WHO) (Good manufacturing practices[1], Good storage and distribution practices[2], MQAS[3], etc.) or of the countries with a high level of regulation (Belgian guidelines, E.U., U.S.A, etc.)[4]. **Reasonable risk taking (with non-negotiable minimum guarantees) and reasoned risk taking** (owing to the tools provided by the WHO[5] or equivalent[6]) is authorized for purchasing, storing and distribution activities carried out in the countries with a low level of regulation.

b. Measurable[7] = **providing proof of the acceptable quality**, e.g. by using the tools provided by the WHO[8] or equivalent, or by **making a risk analysis and management**. For more details we refer to the sections below.

2. Purchasing criteria :

The quality of the pharmaceutical products purchased with Belgian Official Development Aid **must be acceptable and measurable according to the quality criteria mentioned above.**

Three specific cases are possible :

a. **The purchase takes place via the supply system of the partner country** (often called regional/national purchasing offices), **provided that the quality is assured** on the basis of the quality criteria mentioned above. Proof of an acceptable and measurable quality or a risk analysis(and a risk management) is must be provided.

b. If the supply system of the partner country does not offer a sufficient quality guarantee (= « grey zone »), high-quality pharmaceutical products can be purchased directly by the actor involved in the implementation, who has to adopt a pre-qualification policy for the suppliers, according to the quality criteria mentioned above. **He must respect the applicable rules for public procurement, whereas the specification note of each tender must stipulate that only suppliers who guarantee the quality of their pharmaceutical products are allowed to compete.** Proof of an acceptable and measurable quality must be provided. Two specific cases are possible:

    i. **Import** quality assured pharmaceutical products (in so far as the recipient country allows it) following the quality criteria mentioned above : **pharmaceutical products pre-qualified** by the WHO[9] (mainly for HIV/AIDS, tuberculosis, malaria and certain vaccines) or by the Global Fund to Fight AIDS, Tuberculosis and Malaria[10], or registered by a country with a high level of regulation such as Belgium, or equivalent[11] [12];

    ii. **Purchasing via international procurement agencies/suppliers who have been audited and evaluated** according to the quality criteria of the WHO : list of the Humanitarian Procurement Centers (HPC) accredited by the European Commission (DG ECHO)[13], or list of the audited suppliers in the QUAMED database for its members, or equivalent.

c. Should the quality on the level of the local/national procurement circuit not be sufficiently guaranteed AND should it be impossible to import, the actors involved in the implementation undertake not to purchase, store or distribute pharmaceutical products there.

3. Criteria for storage and distribution :

Pharmaceutical products purchased with Belgian Official Development Aid **must be stored and distributed so as to maintain an acceptable and measurable quality in accordance with the quality criteria mentioned above.**

Two specific cases are possible :

a. **The storage and distribution take place via the supply system of the partner country** (often called regional/national purchasing offices), **provided that the quality is ensured** on the basis of the quality criteria mentioned above. Proof of the acceptable and measurable quality demonstrating respect for the **Good Storing and Distribution Practices** developed by the WHO or a reasonable risk analysis (with non-negotiable minimum guarantees) and a reasoned risk analysis (and a risk management) is must be provided.

b. If the supply system of the partner country does not offer a sufficient quality guarantee (= « grey zone »), **the storage and distribution of high-quality pharmaceutical products can be done directly by the actor involved in the implementation, who has to respect the rules of the host country**. Proof of acceptable and measurable quality must be provided : for Belgium, respect of the European directive on Good distribution practices[14].

4. Criteria for contributing to strengthening local capacities :

The implementing actors are committed to strengthening local capacities of the supply system of the partner country aimed to guarantee the quality of pharmaceutical products, since the start of the programme/project.

Different cases are possible : capacity building for pre-qualification of suppliers, purchases, storage and distribution of the local/national supply system of the partner country.

***In witness whereof*,** the undersigned, being duly authorized thereto, have signed this Commitment.

Alexander De Croo

Minister of Development Cooperation

Signature :

Name of the organisation :

Name and function of the signatory:

Signature :

Name of the organisation :

Name and function of the signatory:

Signature :

Name of the organisation :

Name and function of the signatory:

Signature:

[1] WHO Technical Report Series No. 986, 2014 ; Annex 2 – WHO good manufacturing practices (GMP) for pharmaceutical products: main principles ; http://www.who.int/medicines/areas/quality_safety/quality_assurance/expert_committee/trs_986/en/

[2] WHO Technical Report Series, No. 957, 2010 ; Annex 5 - WHO good distribution practices (GDP) for pharmaceutical products ; http://www.who.int/medicines/areas/quality_safety/quality_assurance/GoodDistributionPracticesTRS957Annex5.pdf

[3] WHO Technical Report Series No. 986, 2014 ; Annexe 3- Système modèle d'assurance de la qualité pour agences d'approvisionnement ; http://www.who.int/medicines/areas/quality_safety/quality_assurance/Annexe3-F.pdf

[4] WHO Technical Report Series 961 ; Annex 10 - Autorité de régulation stricte. Il s'agit de l'U.E, Japon, U.S.A, Canada, Australie, Suisse, Islande, Liechtenstein, Norvège.

[5] WHO Technical Report Series No. 986, 2014 ; Annex 3 - Model quality assurance system for procurement agencies - Appendix 6 - Interagency finished pharmaceutical product questionnaire based on the model quality assurance system for procurement agencies ; http://apps.who.int/medicinedocs/documents/s21464en/s21464en.pdf

[6] Tools and services provided by QUAMED for its members; http://www.quamed.be
Tools and services provided by the Inter-Agencies Group.

[7] WHO/EMP/QSM/2010.4, Assessment of medicines regulatory systems in sub-Saharan African countries: an overview of findings from 26 assessment reports ; Genève, 2010.

[8] WHO Technical Report Series 996, 2016; Annex 7 - Guidelines on the conduct of surveys of the quality of medicines; http://www.who.int/medicines/publications/pharmprep/WHO_TRS_996_annex07.pdf?ua=1

[9] WHO Pre-Qualification Programme; http://apps.who.int/prequal/default.htm ; http://www.who.int/immunization_standards/vaccine_quality/PQ_vaccine_list_en/en/

[10] http://www.theglobalfund.org/en/healthproducts/qualityassurance/pharmaceutical/

[11] MSF Qualification Scheme; http://www.msf.org/en/article/msf-qualification-scheme

[12] Approved and Tentatively Approved Antiretrovirals in Association with the President's Emergency Plan (PEPFAR);

http://www.fda.gov/InternationalPrograms/PEPFAR/ucm119231.htm

[13] Liste des Centrales d'Achat Humanitaires (CAH/HPC) accréditées par la Commission Européenne (DG ECHO) disponible sur : http://ec.europa.eu/echo/files/partners/humanitarian_aid/HPC-register_fr.pdf. Il s'agit par exemple en 2016 d'ASRAMES en République Démocratique du Congo, CHMP au Kenya, MSF Supply en Belgique, UNICEF SD, IFRC-GLS, IDA Foundation, etc.

[14] Commission Européenne ; Lignes directrices du 5 novembre 2013 concernant les bonnes pratiques de distribution en gros des médicaments à usage humain ; http://ec.europa.eu/health/files/eudralex/vol-1/2013_c343_01/2013_c343_01_fr.pdf
